# Supplementary material for: Global Dermatophyte Infections Linked to Human and Animal Health: A Scoping Review
Source: Microorganisms. 2025 Mar 3;13(3):575. doi: 10.3390/microorganisms13030575 (PMC11945039; doi:10.3390/microorganisms13030575)
Supplement: Supplementary file 1 [file microorganisms-13-00575-s001.zip › microorganisms-3486806-supplementary.pdf]

Global dermatophyte infections linked to human and animal health

Aditya K. Gupta, Tong Wang, Susmita, Mesbah Talukder, Wayne L. Bakotic

Microorganisms

Supplementary Materials

Figure S1. PRISMA flow chart. 2
Supplementary S1: List of included studies. 3

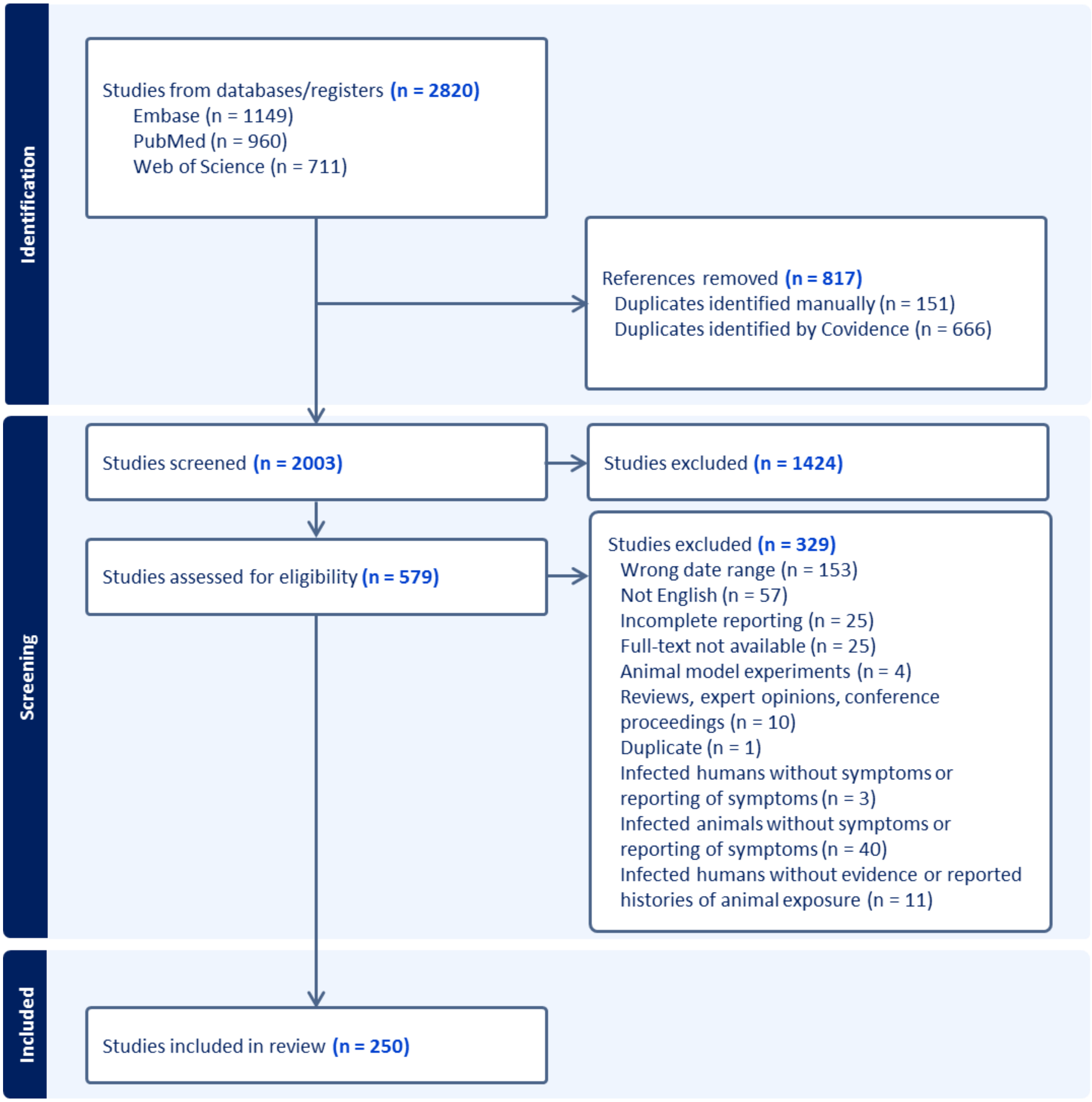

Figure S1. PRISMA flow chart.

Supplementary S1: List of included studies.

1. Abarca, M.L.; Castellá, G.; Martorell, J.; Cabañes, F.J. Trichophyton Erinacei in Pet Hedgehogs in Spain: Occurrence and Revision of Its Taxonomic Status. *Med. Mycol.* **2017**, *55*, 164–172, doi:10.1093/mmy/myw057.

2. Abd-Elmegeed, M.; El-Mekkawi, M.; El-Diasty, E.; Fawzi, E. Dermatophytosis among Ruminants in Egypt: The Infection Rate, Identification and Comparison between Microscopic, Cultural and Molecular Methods. *Zagazig Vet. J.* **2020**, *48*, 116–127, doi:10.21608/zvjz.2019.16779.1081.

3. Abdullah, T.K.; Wadee, S.A.; Owain, M.S. Isolation, Diagnosis and Incidence of Ringworm in Cattle in Salah Al-Din Governorate. *Vet. Pract.* **2021**, *22*, 62–64.

4. Aboueisha, A.M.; El-Mahallawy, H. Public Health Significance of Dermatophytes in Ismailia and Port Said Provinces, Egypt. *Med. Mycol. J.* **2013**, *54*, 123–129, doi:10.3314/mmj.54.123.

5. Adesiji, Y.O.; Oluwayelu, D.O.; Aiyedun, J.O. Prevalence and Risk Factors Associated with Canine Dermatophytoses among Dogs in Kwara and Osun States, Nigeria. *African J. Clin. Exp. Microbiol.* **2023**, *24*, 195–203, doi:10.4314/ajcem.v24i2.9.

6. Afshar, P.; Hedayati, M.T.; Aslani, N.; Khodavaisy, S.; Babamahmoodi, F.; Mahdavi, M.R.; Dolatabadi, S.; Badali, H. First Autochthonous Coinfected Anthrax in an Immunocompetent Patient. *Case Rep. Med.* **2015**, *2015*, 1–6, doi:10.1155/2015/325093.

7. Aghamirian, M.R.; Ghiasian, S.A. Dermatophytes as a Cause of Epizoonoses in Dairy Cattle and Humans in Iran: Epidemiological and Clinical Aspects. *Mycoses* **2011**, *54*, e52–e56, doi:10.1111/j.1439-0507.2009.01832.x.

8. Agnetti, F.; Ciavarella, R.; Cruciani, D.; Epidanio, E.M.; Golinelli, D.; Papa, P.; Sgariglia, E.; Valentini, A.; Crotti, S. Ringworm by Trichophyton Erinacei in Calves: Description of Two Italian Outbreaks. *Large Anim. Rev.* **2020**, *26*, 141–143.

9. Agnetti, F.; Righi, C.; Scoccia, E.; Felici, A.; Crotti, S.; Moretta, I.; Moretti, A.; Maresca, C.; Troiani, L.; Papini, M. Trichophyton Verrucosum Infection in Cattle Farms of Umbria (Central Italy) and Transmission to Humans. *Mycoses* **2014**, *57*, 400–405, doi:10.1111/myc.12174.

10. Ahdy, A.M.; Sayed-Ahmed, M.Z.; Younis, E.E.; Baraka, H.N.; El-khodery, S.A. Prevalence and Potential Risk Factors of Dermatophytosis in Arabian Horses in Egypt. *J. Equine Vet. Sci.* **2016**, *37*, 71–76, doi:10.1016/j.jevs.2015.12.008.

11. Akbarmehr, J. The Prevalence of Cattle Ringworm in Native Dairy Farms of Sarab City (East Azarbayjan Province), Iran. *African J. Microbiol. Res.* **2011**, *5*, 1268–1271, doi:10.5897/ajmr11.001.

12. Alhasan, D.A.; Al-Abedi, H.F.; Hussien, T.J.; Mohammad Ali, A.Q. Morphological Detection of Dermatophytes Isolated from Cattle in Wasit Province. *Iraqi J. Vet. Sci.* **2022**, *36*, 167–172, doi:10.33899/ijvs.2022.135833.2530.

13. Allizond, V.; Tullio, V.; Cuffini, A.M.; Roana, J.; Scalas, D.; Marra, E.S.; Piersigilli, G.; Merlino, C.; Mandras, N.; Banche, G. Advances in Microbiology, Infectious Diseases and Public Health: Fungal Occurrence in the Hair and Skin of Symptomatic Pets in Turin, Italy. *Adv. Exp. Med. Biol.* **2016**, *897*, doi:10.1007/5584_2015_5004.

14. Almuzaini, A.M.; Osman, S.A.; Saeed, E.M.A. An Outbreak of Dermatophytosis in Camels (Camelus Dromedaríus) at Qassim Region, Central of Saudi Arabia. *J. Appl. Anim. Res.* **2016**, *44*, 126–129, doi:10.1080/09712119.2015.1021806.

15. Ansari, S.; Ahmadi, B.; Tabatabaeifar, S.N.; Hedayati, M.T.; Javidnia, J.; Taghizadeh Armaki, M.; Shokoohi, G.R.; Rezaei-Matehkolaei, A. Familial Cases of Trichophyton Benhamiae Infection Transmitted from a Guinea Pig in Iran. *Mycopathologia* **2021**, *186*, 119–125, doi:10.1007/s11046-020-00513-1.

16. Apprich, V.; Spergser, J.; Rosengarten, R.; Hinterhofer, C.; Stanek, C. Scanning Electron Microscopy and Fungal Culture of Hoof Horn from Horses Suffering from Onychomycosis. *Vet. Dermatol.* **2010**, *21*, 335–340, doi:10.1111/j.1365-3164.2009.00864.x.

17. Arabatzis, M.; Kyprianou, M.; Velegraki, A.; Makri, A.; Voyatzi, A. Microsporum Canis Antifungal Susceptibilities: Concerns Regarding Their Clinical Predictability. *Int. J. Antimicrob. Agents* **2010**, *36*, 385–386, doi:10.1016/j.ijantimicag.2010.06.032.

18. Bartosch, T.; Frank, A.; Günther, C.; Uhrlaß, S.; Heydel, T.; Nenoff, P.; Baums, C.G.; Schrödl, W. Trichophyton Benhamiae and T. Mentagrophytes Target Guinea Pigs in a Mixed Small Animal Stock. *Med. Mycol. Case Rep.* **2019**, *23*, 37–42, doi:10.1016/j.mmcr.2018.11.005.

19. Begum, J.; Kumar, R. Prevalence of Dermatophytosis in Animals and Antifungal Susceptibility Testing of Isolated Trichophyton and Microsporum Species. *Trop. Anim. Health Prod.* **2021**, *53*, doi:10.1007/s11250-020-02476-3.

20. Berlin, M.; Kupsch, C.; Ritter, L.; Stoelcker, B.; Heusinger, A.; Gräser, Y. German-Wide Analysis of the Prevalence and the Propagation Factors of the Zoonotic Dermatophyte Trichophyton Benhamiae. *J. Fungi* **2020**, *6*, 1–11, doi:10.3390/jof6030161.

21. Bernhardt, A.; Von Bomhard, W.; Antweiler, E.; Tintelnot, K. Molecular Identification of Fungal Pathogens in Nodular Skin Lesions of Cats. *Med. Mycol.* **2015**, *53*, 132–144, doi:10.1093/mmy/myu082.

22. Bescrovaine, J.D.O.; Warth, J.F.G.; De Souza, C.; Benoni, V.W.; Baja, F.; Schneider, G.X.; Vicente, V.A.; De Hoog, G.S.; Queiroz-Telles, F. Nannizzia Species Causing Dermatophytosis in Cats and Dogs: First Report of Nannizzia Incurvata as an Etiological Agent in Brazil. *Med. Mycol.* **2023**, *61*, doi:10.1093/mmy/myad105.

23. Bianchi, M. V; Laisse, C.J.M.; Vargas, T.P.; Wouters, F.; Boabaid, F.M.; Pavarini, S.P.; Ferreiro, L.; Driemeier, D. Intra-Abdominal Fungal Pseudomycetoma in Two Cats. *Rev. Iberoam. Micol.* **2017**, *34*, 112–115, doi:10.1016/j.riam.2016.10.001.

24. Borges-Costa, J.; Martins, M. da L. Trichophyton Erinacei Skin Infection after Recreational Exposure to an Elephant in Southeast Asia. *Pathog. Glob. Health* **2014**, *108*,

58–59, doi:10.1179/2047773213Y.0000000117.

25. Brasch, J.; Lögering, B.; Gräser, Y. Tinea Capitis Caused by Trichophyton Equinum. *Acta Derm. Venereol.* **2009**, *89*, 204–205, doi:10.2340/00015555-0596.

26. Brosh-Nissimov, T.; Ben-Ami, R.; Astman, N.; Malin, A.; Baruch, Y.; Galor, I. An Outbreak of Microsporum Canis Infection at a Military Base Associated with Stray Cat Exposure and Person-to-Person Transmission. *Mycoses* **2018**, *61*, 472–476, doi:10.1111/myc.12771.

27. Budihardja, D.; Freund, V.; Mayser, P. Widespread Erosive Tinea Corporis by Arthroderma Benhamiae in a Renal Transplant Recipient: Case Report. *Mycoses* **2010**, *53*, 530–532, doi:10.1111/j.1439-0507.2009.01736.x.

28. Cabral, F. V; Sellera, F.P.; Ribeiro, M.S. Methylene Blue-Mediated Antimicrobial Photodynamic Therapy for Canine Dermatophytosis Caused by Microsporum Canis: A Successful Case Report with 6 Months Follow-Up. *Photodiagnosis Photodyn. Ther.* **2021**, *36*, 1–3, doi:10.1016/j.pdpdt.2021.102602.

29. Cafarchia, C.; Camarda, A.; Coccioli, C.; Figueredo, L.A.; Circella, E.; Danesi, P.; Capelli, G.; Otranto, D. Epidemiology and Risk Factors for Dermatophytoses in Rabbit Farms. *Med. Mycol.* **2010**, *48*, 975–980, doi:10.3109/13693781003652620.

30. Cafarchia, C.; Figueredo, L.A.; Coccioli, C.; Camarda, A.; Otranto, D. Enzymatic Activity of Microsporum Canis and Trichophyton Mentagrophytes from Breeding Rabbits with and without Skin Lesions. *Mycoses* **2012**, *55*, 45–49, doi:10.1111/j.1439-0507.2010.01997.x.

31. Cafarchia, C.; Weigl, S.; Figueredo, L.A.; Otranto, D. Molecular Identification and Phylogenesis of Dermatophytes Isolated from Rabbit Farms and Rabbit Farm Workers. *Vet. Microbiol.* **2012**, *154*, 395–402, doi:10.1016/j.vetmic.2011.07.021.

32. Çam, Y.; Koç, A.N.; Silici, S.; Günes, V.; Buldu, H.; Onmaz, A.C.; Kasap, F.F. Treatment of Dermatophytosis in Young Cattle with Propolis and Whitfield’s Ointment. *Vet. Rec.* **2009**, *165*, 57–58, doi:10.1136/vetrec.165.2.57.

33. Capoor, M.R.; Sharma, S.; Goenka, S.; Das, S.; Rudramurthy, S.M.; Khunger, N.; kamra, N. “Tinea Capitis Caused by Microsporum Canis: A Case Study of Three Family Members in India, a Non-Endemic Region. *Indian J. Med. Microbiol.* **2024**, *50*, 100621, doi:10.1016/j.ijmmb.2024.100621.

34. Chah, K.F.; Majiagbe, K.A.; Kazeem, H.M.; Ezeanyika, O.; Agbo, I.C. Dermatophytes from Skin Lesions of Domestic Animals in Nsukka, Enugu State, Nigeria. *Vet. Dermatol.* **2012**, *23*, 522–525, doi:10.1111/j.1365-3164.2012.01089.x.

35. Cho, J.; Park, C.; Park, J.; Yoon, J.S. Case Report: Dermatophytic Pseudomycetoma in a Domestic Korean Short Hair Cat Treated with Intralesional Injection of Amphotericin B and Oral Terbinafine Administration. *Front. Vet. Sci.* **2024**, *11*, 1–5, doi:10.3389/fvets.2024.1402691.

36. Choi, E.; Huang, J.; Chew, K.L.; Jaffar, H.; Tan, C. Pustular Tinea Manuum from Trichophyton Erinacei Infection. *JAAD Case Reports* **2018**, *4*, 518–520, doi:10.1016/j.jdcr.2018.01.019.

37. Chollet, A.; Wespi, B.; Roosje, P.; Unger, L.; Venner, M.; Goepfert, C.; Monod, M. An Outbreak of Arthroderma Vanbreuseghemii Dermatophytosis at a Veterinary School Associated with an Infected Horse. *Mycoses* **2015**, *58*, 233–238, doi:10.1111/myc.12301.

38. Chung, T.; Ph, D.; Kim, E.; Choi, U.S.; Ph, D. MULTIORGAN FUNGAL INFECTION CAUSED BY MICROSPORUM CANIS IN A GREEN IGUANA (IGUANA IGUANA) Author (s): Tae-Ho Chung , Eun-Ju Kim and Ul Soo Choi Source : Journal of Zoo and Wildlife Medicine , June 2014 , Vol . 45 , No . 2 (June 2014), Published by : American Association of Zoo Veterinarians Stable URL : Https://Www.Jstor.Org/Stable/24551110 MULTIORGAN FUNGAL INFECTION CAUSED BY MICROSPORUM. **2014**, *45*, 393–396.

39. Chupia, V.; Ninsuwon, J.; Piyarungsri, K.; Sodarat, C.; Prachasilchai, W.; Suriyasathaporn, W.; Pikulkaew, S. Prevalence of Microsporum Canis from Pet Cats in Small Animal Hospitals, Chiang Mai, Thailand. *Vet. Sci.* **2022**, *9*, 21, doi:10.3390/vetsci9010021.

40. Clothier, K.A.; Watson, K.D.; Mete, A.; Giannitti, F.; Anderson, M.; Munk, B.; McMillin, S.; Clifford, D.L.; Rudd, J.; Shirkey, N.; et al. Generalized Dermatophytosis Caused by Trichophyton Equinum in 8 Juvenile Black Bears in California. *J. Vet. Diagnostic Investig.* **2022**, *34*, 279–283, doi:10.1177/10406387211061143.

41. Čmoková, A.; Rezaei-Matehkolaei, A.; Kuklová, I.; Kolařík, M.; Shamsizadeh, F.; Ansari, S.; Gharaghani, M.; Miňovská, V.; Najafzadeh, M.J.; Nouripour-Sisakht, S.; et al. Discovery of New Trichophyton Members, T. Persicum and T. Spiraliforme Spp. Nov., as a Cause of Highly Inflammatory Tinea Cases in Iran and Czechia. *Microbiol. Spectr.* **2021**, *9*, 1–20, doi:10.1128/Spectrum.00284-21.

42. Colombo, S.; Scarampella, F.; Ordeix, L.; Roccabianca, P. Dermatophytosis and Papular Eosinophilic/Mastocytic Dermatitis (Urticaria Pigmentosa-like Dermatitis) in Three Devon Rex Cats. *J. Feline Med. Surg.* **2012**, *14*, 498–502, doi:10.1177/1098612X12440761.

43. Concha, M.; Nicklas, C.; Balcells, E.; Guzmán, A.M.; Poggi, H.; León, E.; Fich, F. The First Case of Tinea Faciei Caused by Trichophyton Mentagrophytes Var. Erinacei Isolated in Chile. *Int. J. Dermatol.* **2012**, *51*, 283–285, doi:10.1111/j.1365-4632.2011.04995.x.

44. Cornegliani, L.; Persico, P.; Colombo, S. Canine Nodular Dermatophytosis (Kerion): 23 Cases. *Vet. Dermatol.* **2009**, *20*, 185–190, doi:10.1111/j.1365-3164.2009.00749.x.

45. Courtellemont, L.; Chevrier, S.; Degeilh, B.; Belaz, S.; Gangneux, J.P.; Robert-Gangneux, F. Epidemiology of Trichophyton Verrucosum Infection in Rennes University Hospital, France: A 12-Year Retrospective Study. *Med. Mycol.* **2017**, *55*, 720–724, doi:10.1093/mmy/myw142.

46. Cruciani, D.; Papini, M.; Broccatelli, S.; Agnetti, F.; Spina, S.; Natalini, Y.; Crotti, S. Presumptive Zoonotic Kerion by Nannizzia Gypsea: Case Report. *Front. Vet. Sci.* **2021**, *8*, 1–5, doi:10.3389/fvets.2021.718766.

47. Cukierman, E.; Camargo, T.Z.S.; Millan, L.P.B.; Freire, M.R. de M.; Carneiro, L.F.M.; Waksman, R.D. Alopecia and Pet: A Case Report. *Einstein (Sao Paulo).* **2022**, *20*,

eRC6881, doi:10.31744/einstein_journal/2022RC6881.

48. Czaika, V.A. Effective Treatment of Tinea Corporis Due to Trichophyton Mentagrophytes with Combined Isoconazole Nitrate and Diflucortolone Valerate Therapy. *Mycoses* **2013**, *56*, 30–32, doi:10.1111/myc.12068.

49. Czaika, V.A. Misdiagnosed Zoophile Tinea Faciei and Tinea Corporis Effectively Treated with Isoconazole Nitrate and Diflucortolone Valerate Combination Therapy. *Mycoses* **2013**, *56*, 26–29, doi:10.1111/myc.12057.

50. da Costa, F.V.A.; Farias, M.R.; Bier, D.; de Andrade, C.P.; de Castro, L.A.; da Silva, S.C.; Ferreiro, L. Genetic Variability in Microsporum Canis Isolated from Cats, Dogs and Humans in Brazil. *Mycoses* **2013**, *56*, 582–588, doi:10.1111/myc.12078.

51. da Cunha, M.M.; Capote-Bonato, F.; Capoci, I.R.G.; Bonato, D.V.; Ghizzi, L.G.; Paiva-Lima, P.; Baeza, L.C.; Svidzinski, T.I.E. Epidemiological Investigation and Molecular Typing of Dermatophytosis Caused by Microsporum Canis in Dogs and Cats. *Prev. Vet. Med.* **2019**, *167*, 39–45, doi:10.1016/j.prevetmed.2019.03.019.

52. Dalis, J.S.; Kazeem, H.M.; Kwaga, J.K.P.; Kwanashie, C.N. Prevalence and Distribution of Dermatophytosis Lesions on Cattle in Plateau State, Nigeria. *Vet. World* **2019**, *12*, 1484–1490, doi:10.14202/vetworld.2019.1484-1490.

53. de Freitas, R.S.; de Freitas, T.H.P.; Siqueira, L.P.M.; Gimenes, V.M.F.; Benard, G. First Report of Tinea Corporis Caused by Arthroderma Benhamiae in Brazil. *Braz. J. Microbiol.* **2019**, *50*, 985–987, doi:10.1007/s42770-019-00141-y.

54. Demitsu, T.; Yamada, T.; Umemoto, N.; Kakurai, M.; Maeda, T.; Harada, K.; Kawase, M. Disseminated Dermatophytosis Due to Nannizzia Gypsea (Microsporum Gypseum) in an Elderly Patient. *J. Dermatol.* **2019**, *46*, e169–e170, doi:10.1111/1346-8138.14712.

55. DeTar, L.G.; Dubrovsky, V.; Scarlett, J.M. Descriptive Epidemiology and Test Characteristics of Cats Diagnosed with Microsporum Canis Dermatophytosis in a Northwestern US Animal Shelter. *J. Feline Med. Surg.* **2019**, *21*, 1198–1205, doi:10.1177/1098612X19825519.

56. Dewal, V.S.; Chahar, A.; Tuteja, F.C.; Tanwar, R.K.; Singh, A.P.; Rathore, N.S.; Savita Dermatophytosis in Dromedary Camel (Camelus Dromedarius). *Vet. Pract.* **2017**, *18*, 233–236.

57. Dogo, J.; Afegbua, S.L.; Dung, E.C. Prevalence of Tinea Capitis among School Children in Nok Community of Kaduna State, Nigeria. *J. Pathog.* **2016**, *2016*, 1–6, doi:10.1155/2016/9601717.

58. Dong, C.; Angus, J.; Scarampella, F.; Neradilek, M. Evaluation of Dermoscopy in the Diagnosis of Naturally Occurring Dermatophytosis in Cats. *Vet. Dermatol.* **2016**, *27*, 275-e65, doi:10.1111/vde.12333.

59. d’Ovidio, D.; Santoro, D. Survey of Zoonotic Dermatoses in Client-Owned Exotic Pet Mammals in Southern Italy. *Zoonoses Public Health* **2015**, *62*, 100–104, doi:10.1111/zph.12100.

60. Drira, I.; Neji, S.; Hadrich, I.; Sellami, H.; Makni, F.; Ayadi, A. Tinea Manuum Due to Trichophyton Erinacei from Tunisia. *J. Mycol. Med.* **2015**, *25*, 200–203, doi:10.1016/j.mycmed.2015.05.001.

61. Drouot, S.; Mignon, B.; Fratti, M.; Roosje, P.; Monod, M. Pets as the Main Source of Two Zoonotic Species of the Trichophyton Mentagrophytes Complex in Switzerland, Arthroderma Vanbreuseghemii and Arthroderma Benhamiae. *Vet. Dermatol.* **2009**, *20*, 13–18, doi:10.1111/j.1365-3164.2008.00691.x.

62. Duangkaew, L.; Larsuprom, L.; Kasondorkbua, C.; Chen, C.; Chindamporn, A. Cutaneous Blastomycosis and Dermatophytic Pseudomycetoma in a Persian Cat from Bangkok, Thailand. *Med. Mycol. Case Rep.* **2017**, *15*, 12–15, doi:10.1016/j.mmcr.2017.01.001.

63. Duarte ER, Oliveiraa NJF, Medeirosb AO, R.C. and F.-F.E. Yeasts Isolated from Beef Heifers with Ringworm Levaduras Aisladas de Terneras de Carne Con Dermatofitosis. *Arch Med Vet* **2013**, *45*, 71–75.

64. El Damaty, H.M.; Tartor, Y.H.; Mahmmod, Y.S. Species Identification, Strain Differentiation, and Antifungal Susceptibility of Dermatophyte Species Isolated From Clinically Infected Arabian Horses. *J. Equine Vet. Sci.* **2017**, *59*, 26–33, doi:10.1016/j.jevs.2017.08.019.

65. Errichetti, E.; Pietkiewicz, P.; Bhat, Y.J.; Salwowska, N.; Szlązak, P.; Stinco, G. Diagnostic Accuracy of Ultraviolet-Induced Fluorescence Dermoscopy in Non-Neoplastic Dermatoses (General Dermatology): A Multicentric Retrospective Comparative Study. *J. Eur. Acad. Dermatology Venereol.* **2024**, 1–12, doi:10.1111/jdv.19795.

66. Farag, A.G.A.; Hammam, M.A.; Ibrahem, R.A.; Mahfouz, R.Z.; Elnaidany, N.F.; Qutubuddin, M.; Tolba, R.R.E. Epidemiology of Dermatophyte Infections among School Children in Menoufia Governorate, Egypt. *Mycoses* **2018**, *61*, 321–325, doi:10.1111/myc.12743.

67. Figueredo, L.A.; Cafarchia, C.; Otranto, D. Geotrichum Candidum as Etiological Agent of Horse Dermatomycosis. *Vet. Microbiol.* **2011**, *148*, 368–371, doi:10.1016/j.vetmic.2010.09.025.

68. Fratti, M.; Bontems, O.; Salamin, K.; Guenova, E.; Monod, M. Survey on Dermatophytes Isolated from Animals in Switzerland in the Context of the Prevention of Zoonotic Dermatophytosis. *J. Fungi* **2023**, *9*, doi:10.3390/jof9020253.

69. Gamage, H.; Sivanesan, P.; Hipler, U.C.; Elsner, P.; Wiegand, C. Superficial Fungal Infections in the Department of Dermatology, University Hospital Jena: A 7-Year Retrospective Study on 4556 Samples from 2007 to 2013. *Mycoses* **2020**, *63*, 558–565, doi:10.1111/myc.13077.

70. Gangil, R.; Dutta, P.; Tripathi, R.; Singathia, R.; Lakhotia, R.L. Incidence of Dermatophytosis in Canine Cases Presented at Apollo Veterinary College, Rajashtan, India. *Vet. World* **2012**, *5*, 682–684, doi:10.5455/vetworld.2012.682-684.

71. Gergovska, M.; Hitova, M.; Manuelyan, K.; Kazandjieva, J. Acute Inflammatory Tinea Manuum Caused by Nannizzia Gypsea Transmitted by an African Pygmy Hedgehog. *J.*

Eur. Acad. Dermatology Venereol. **2024**, *38*, e767–e768, doi:10.1111/jdv.19874.

72. Giner, J.; Bailey, J.; Juan-Sallés, C.; Joiner, K.; Martínez-Romero, E.G.; Oster, S. Dermatophytic Pseudomycetomas in Two Ferrets (Mustela Putorius Furo). *Vet. Dermatol.* **2018**, *29*, 452-e154, doi:10.1111/vde.12673.

73. Gnat, S.; Łagowski, D.; Dyląg, M.; Nowakiewicz, A. European Hedgehogs (Erinaceus Europaeus L.) as a Reservoir of Dermatophytes in Poland. *Microb. Ecol.* **2022**, *84*, 363–375, doi:10.1007/s00248-021-01866-w.

74. Gnat, S.; Łagowski, D.; Nowakiewicz, A.; Dyląg, M. Tinea Corporis Caused by Trichophyton Equinum Transmitted from Asymptomatic Dogs to Two Siblings. *Brazilian J. Microbiol.* **2020**, *51*, 1433–1438, doi:10.1007/s42770-019-00204-0.

75. Gnat, S.; Łagowski, D.; Nowakiewicz, A.; Osińska, M.; Kopiński, Ł. Population Differentiation, Antifungal Susceptibility, and Host Range of Trichophyton Mentagrophytes Isolates Causing Recalcitrant Infections in Humans and Animals. *Eur. J. Clin. Microbiol. Infect. Dis.* **2020**, *39*, 2099–2113, doi:10.1007/s10096-020-03952-2.

76. Gnat, S.; Łagowski, D.; Nowakiewicz, A.; Trościańczyk, A.; Zięba, P. Infection of Trichophyton Verrucosum in Cattle Breeders, Poland: A 40-Year Retrospective Study on the Genomic Variability of Strains. *Mycoses* **2018**, *61*, 681–690, doi:10.1111/myc.12791.

77. Gnat, S.; Łagowski, D.; Nowakiewicz, A.; Zięba, P. Tinea Corporis by Microsporum Canis in Mycological Laboratory Staff: Unexpected Results of Epidemiological Investigation. *Mycoses* **2018**, *61*, 945–953, doi:10.1111/myc.12832.

78. Gnat, S.; Nowakiewicz, A.; Lagowski, D.; Czyk, A.T.; Zieba, P. Multiple-Strain Trichophyton Mentagrophytes Infection in a Silver Fox (Vulpes Vulpes) from a Breeding Farm. *Med. Mycol.* **2019**, *57*, 171–180, doi:10.1093/MMY/MYY011.

79. Gordon, E.; Idle, A.; DeTar, L. Descriptive Epidemiology of Companion Animal Dermatophytosis in a Canadian Pacific Northwest Animal Shelter System. *Can. Vet. J. = La Rev. Vet. Can.* **2020**, *61*, 763–770.

80. Guebeli, A.; Honigmann, P.; Mertz, K.; Willi, N.; Claas, G.J.; Keller, M. A Rare Case of Cutaneous Trichophyton Verrucosum of the Forearm in a 51-Year-Old Cattle Farmer. *J. Hand Surg. Am.* **2021**, *46*, 1128.e1-1128.e4, doi:10.1016/j.jhsa.2020.12.007.

81. Guo, Y.; Ge, S.; Luo, H.; Rehman, A.; Li, Y.; He, S. Occurrence of Trichophyton Verrucosum in Cattle in the Ningxia Hui Autonomous Region, China. *BMC Vet. Res.* **2020**, *16*, 1–9, doi:10.1186/s12917-020-02403-6.

82. Hackworth, C.E.; Eshar, D.; Nau, M.; Bagladi-Swanson, M.; Andrews, G.A.; Carpenter, J.W. Diagnosis and Successful Treatment of a Potentially Zoonotic Dermatophytosis Caused by Microsporum Gypseum in a Zoo-Housed North American Porcupine (Erethizon Dorsatum). *J. Zoo Wildl. Med.* **2017**, *48*, 549–553, doi:10.1638/2016-0097R1.1.

83. Hameed, K.; Ch, F.R.; Nawaz, M.A.; Naqvi, S.M.S.; Gräser, Y.; Kupsch, C.; Pasquetti, M.; Rossi, L.; Molinar Min, A.R.; Tizzani, P.; et al. Trichophyton Verrucosum Infection in Livestock in the Chitral District of Pakistan. *J. Infect. Dev. Ctries.* **2017**, *11*, 326–333,

doi:10.3855/jidc.7925.

84. Hariu, M.; Watanabe, Y.; Shimada, D.; Imai, H.; Takano, K.; Kamioka, Y.; Seki, M. A Household Microsporum Canis Dermatophytosis Suggested by Matrix-Assisted Laser Desorption/Ionization Time-Of-Flight Mass Spectrometry Analysis. *Am. J. Case Rep.* **2021**, *22*, 2–5, doi:10.12659/AJCR.930713.

85. Hernandez-Bures, A.; Pieper, J.B.; Bidot, W.A.; O'Dell, M.; Sander, W.E.; Maddox, C.W. Survey of Dermatophytes in Stray Dogs and Cats with and without Skin Lesions in Puerto Rico and Confirmed with MALDI-TOF MS. *PLoS One* **2021**, *16*, 1–10, doi:10.1371/journal.pone.0257514.

86. Hnilica, K.A.; Medleau, L. Evaluation of Topically Applied Enilconazole for the Treatment of Dermatophytosis in a Persian Cattery. *Vet. Dermatol.* **2002**, *13*, 23–28, doi:10.1046/j.0959-4493.2001.00282.x.

87. Hobi, S.; Tam, W.Y.J.; Tse, M.; Nekouei, O.; Chai, Y.; Hill, F.I.; Cheung, E.; Botes, W.; Saulnier-Troff, F.; McDermott, C.T.; et al. Microsporum Canis Causes Cutaneous and Extracutaneous Feline Dermatophytic Pseudomycetomas: Molecular Identification and Clinicopathological Characteristics. *J. Fungi* **2024**, *10*, doi:10.3390/jof10080576.

88. Hsiao, Y.H.; Chen, C.; Han, H.S.; Kano, R. The First Report of Terbinafine Resistance Microsporum Canis from a Cat. *J. Vet. Med. Sci.* 2018, *80*, 898–900.

89. Hsieh, C.W.; Sun, P.L.; Wu, Y.H. Trichophyton Erinacei Infection from a Hedgehog: A Case Report from Taiwan. *Mycopathologia* **2010**, *170*, 417–421, doi:10.1007/s11046-010-9333-2.

90. Hubka, V.; Dobiášová, S.; Dobiáš, R.; Kolařík, M. Microsporum Aenigmaticum Sp. Nov. from M. Gypseum Complex, Isolated as a Cause of Tinea Corporis. *Med. Mycol.* **2014**, *52*, 387–396, doi:10.1093/mmy/myt033.

91. Ilhan, Z.; Karaca, M.; Ekin, I.H.; Solmaz, H.; Akkan, H.A.; Tutuncu, M. Detection of Seasonal Asymptomatic Dermatophytes in Van Cats. *Brazilian J. Microbiol.* **2016**, *47*, 225–230, doi:10.1016/j.bjm.2015.11.027.

92. Ito, A.; Yamada, N.; Kimura, R.; Anzawa, K.; Mochizuki, T.; Yamamoto, O. Tinea Barbae Due to Trichophyton Mentagrophytes Contracted from Calves. *Acta Derm. Venereol.* **2019**, *99*, 925–926, doi:10.2340/00015555-3247.

93. Ivaskiene, M.; Matusevicius, A.P.; Grigonis, A.; Zamokas, G.; Babickaite, L. Efficacy of Topical Therapy with Newly Developed Terbinafine and Econazole Formulations in the Treatment of Dermatophytosis in Cats. *Pol. J. Vet. Sci.* **2016**, *19*, 535–543, doi:10.1515/pjvs-2016-0067.

94. Iznardo, H.; Garcia-Melendo, C.; Berengua, C. Tinea Facei from Trichophyton Benhamiae from a Pet Guinea Pig. *JAMA Dermatology* **2021**, *157*, 1115, doi:10.1001/jamadermatol.2021.2566.

95. Jain, G.K.; Singh, A.P.; Tuteja, F.C.; Marwaha, S.; Sharma, A.; Kachhawa, J.P.; Gupta, S.R.; Gaur, A.K.; Tanwar, J. Study of Dermal Mycosis in Camels (Camelus Dromedarius) in and around Bikaner. *Vet. Pract.* **2019**, *20*, 195–198.

96. Jakubowicz, O.; Łuczkowska, M.; Zaba, R.; Adamski, Z. Tinea Cutis Glabrae: Causes of Diagnostic Challenge. *Postep. Dermatologii i Alergol.* **2014**, *31*, 421–424, doi:10.5114/pdia.2014.40950.

97. Jańczak, D.; Górecki, P.; Maj, A.K. PCR-Based Methods in Detection and Identification of Dermatophytes in Dogs and Cats with Suspected Dermatophytosis in 2021 in Poland. *Pol. J. Vet. Sci.* **2023**, *26*, 629–634, doi:10.24425/pjvs.2023.148282.

98. Jang, M.S.; Park, J. Bin; Jang, J.Y.; Yang, M.H.; Kim, J.H.; Lee, K.H.; Hwangbo, H.; Suh, K.S. Kerion Celsi Caused by Trichophyton Erinacei from a Hedgehog Treated with Terbinafine. *J. Dermatol.* **2017**, *44*, 1070–1071, doi:10.1111/1346-8138.13647.

99. Jarjees, K.I.; Issa, N.A. First Study on Molecular Epidemiology of Dermatophytosis in Cats, Dogs, and Their Companions in the Kurdistan Region of Iraq. *Vet. World* **2022**, *15*, 2971–2978, doi:10.14202/vetworld.2022.2971-2978.

100. Jaspers, G.J.; Werrij, B.G.; Jagtman, B.A.; Loza, B. Severe Kerion Celsi Due to Trichophyton Mentagrophytes: A Case Report. *Acta Paediatr. Int. J. Paediatr.* **2011**, *100*, 183–185, doi:10.1111/j.1651-2227.2011.02256.x.

101. Jiang, Y.; Zhan, P.; Al-Hatmi, A.M.S.; Shi, G.; Wei, Y.; van den Ende, A.H.G.G.; Meis, J.F.; Lu, H.; de Hoog, G.S. Extensive Tinea Capitis and Corporis in a Child Caused by Trichophyton Verrucosum. *J. Mycol. Med.* **2019**, *29*, 62–66, doi:10.1016/j.mycmed.2019.01.007.

102. Jokelainen, P.; Virtala, A.M.K.; Raulo, S.; Kantele, A.; Vapalahti, O.; Kinnunen, P.M. Veterinarians and Zoonotic Pathogens, Infections and Diseases–Questionnaire Study and Case Series, Finland. *Infect. Dis. (Auckl).* **2024**, *56*, 384–392, doi:10.1080/23744235.2024.2313662.

103. Kaneko, T.; Kaneko, M.; Makimura, K. Cluster Analysis of Microsporum Canis Isolated from a Patient with Tinea Corporis and an Infected Cat Based on the DNA Sequences of Nuclear Ribosomal Internal Transcribed Spacer 1. *Mycoses* **2011**, *54*, e867–e869, doi:10.1111/j.1439-0507.2011.02014.x.

104. Kano, R.; Edamura, K.; Yumikura, H.; Maruyama, H.; Asano, K.; Tanaka, S.; Hasegawa, A. Confirmed Case of Feline Mycetoma Due to Microsporum Canis. *Mycoses* **2009**, *52*, 80–83, doi:10.1111/j.1439-0507.2008.01518.x.

105. Kano, R.; Nagata, M.; Suzuki, T.; Watanabe, S.; Kamata, H.; Hasegawa, A. Isolation of Trichophyton Rubrum Var. Raubitschekii from a Dog. *Med. Mycol.* **2010**, *48*, 653–655, doi:10.3109/13693780903403043.

106. Kasmaei, A.; Salimi, A.; Helan, J.A.; Asl, S.A.S.; Katiraee, F. Molecular Study of Feline Dermatophytosis and Toll-like Receptor 2 and 4 Gene Expression in Their Lesions. *Vet. Med. Sci.* **2023**, *9*, 1036–1042, doi:10.1002/vms3.1120.

107. Katiraee, F.; Kosari, Y.K.; Soltani, M.; Shokri, H.; Minooieanhaghighi, M.H. Molecular Identification and Antifungal Susceptibility Patterns of Dermatophytes Isolated from Companion Animals with Clinical Symptoms of Dermatophytosis. *J. Vet. Res.* **2021**, *65*, 175–182, doi:10.2478/jvetres-2021-0020.

108. Keeble, E.J.; Neuber, A.; Hume, L.; Goodman, G. Medical Management of Trichophyton Dermatophytosis Using a Novel Treatment Regimen in L'Hoest's Monkeys (Cercopithecus Lhoesti). *Vet. Rec.* **2010**, *167*, 862–864, doi:10.1136/vr.c5426.

109. Keskin Yilmaz, N.; Baş, B. Superficial Pyoderma in Cats and Dogs: A Retrospective Clinical Study. *Ankara Univ. Vet. Fak. Derg.* **2024**, *71*, 207–213, doi:10.33988/auvfd.1239626.

110. Khalaf, J.M.; Minnat, T.R.; Hussein, R.A. Equine Dermatophytosis: Clinical, Epidemiological and Species Distribution among Horses of Equestrian Club in Baghdad Governorate, Iraq. *Vet. Pract.* **2020**, *21*, 457–463.

111. Kheffache, H.; Seklaoui, N.; Bouchara, J.P.; Boukhemza-Zemmouri, N.; Boukhemza, M. Tinea Capitis at the University Hospital of Tizi-Ouzou, Algeria, and First Isolation of Trichophyton Tonsurans. *J. Mycol. Med.* **2020**, *30*, 101040, doi:10.1016/j.mycmed.2020.101040.

112. Khiewplueang, K.; Leeyaphan, C.; Bunyaratavej, S.; Jirawattanadon, P.; Saengthong-aram, P.; Matthapan, L.; Prasong, W.; Panyawong, C.; Plengpanich, A. Tinea Faciei Clinical Characteristics, Causative Agents, Treatments and Outcomes; a Retrospective Study in Thailand. *Mycoses* **2024**, *67*, 1–7, doi:10.1111/myc.13754.

113. Khosravi, A.R.; Shokri, H.; Rostami, A.; Tamai, I.A.; Erfanmanesh, A.; Memarian, I. Severe Dermatophytosis Due to Trichophyton Mentagrophytes Var. Interdigitale in Flocks of Green Iguanas (Iguana Iguana). *J. Small Anim. Pract.* **2012**, *53*, 286–291, doi:10.1111/j.1748-5827.2011.01217.x.

114. Kim, J.; Tsuchihashi, H.; Hiruma, M.; Kano, R.; Ikeda, S. Tinea Corporis Due to Trichophyton Erinacei Probably Transmitted from a Hedgehog: The Second Case Report from Japan. *Med. Mycol. J.* **2018**, *59*, E77–E79, doi:10.3314/mmj.18-00006.

115. Kirmizigul, A.H.; Otlu, S.; Erkilic, E.E.; Buyuk, F.; Tüfenk, D.Ş.; Gulmez Saglam, A.; Çelebi, Ö.; Akyuz, E. The Mycotic Agent Profile of Calves with Clinical Dermatophytosis Complaints: A Multifactorial Portrait. *Fresenius Environ. Bull.* **2021**, *30*, 12735–12740.

116. Kizerwetter-Świda, M.; Bąk, I.; Biegańska, M.J.; Dembele, K.; Chrobak-Chmiel, D. Chrysosporium Articulatum Mimicking Trichophyton Spp. Infection in a Cat: A Case Presentation and Literature Review. *BMC Vet. Res.* **2024**, *20*, 1–7, doi:10.1186/s12917-024-04185-7.

117. Krajden, S.; Summerbell, R.C.; Datt, A.; Hawke, M.; Scott, J. Monkey Finger Mycology? First Case of Otomycosis Externa Caused by Trichophyton Simii after Encounter with a Monkey. *Med. Mycol. Case Rep.* **2022**, *37*, 17–18, doi:10.1016/j.mmcr.2022.06.001.

118. Kromer, C.; Nenoff, P.; Uhrlaß, S.; Apel, A.; Schön, M.P.; Lippert, U. Trichophyton Erinacei Transmitted to a Pregnant Woman From Her Pet Hedgehogs. *JAMA dermatology* **2018**, *154*, 967–968, doi:10.1001/jamadermatol.2018.1124.

119. Łagowski, D.; Gnat, S.; Dyląg, M.; Nowakiewicz, A. Laboratory Diagnosis and In Vitro Antifungal Susceptibility of Trichophyton Quinckeanum from Human Zoonoses and Cats. *Antibiotics* **2022**, *11*, 1–13, doi:10.3390/antibiotics11060739.

120. Łagowski, D.; Gnat, S.; Nowakiewicz, A.; Osińska, M.; Trościańczyk, A.; Zięba, P. In Search of the Source of Dermatophytosis: Epidemiological Analysis of Trichophyton Verrucosum Infection in Llamas and the Breeder (Case Report). *Zoonoses Public Health* **2019**, *66*, 982–989, doi:10.1111/zph.12648.

121. Łagowski, D.; Gnat, S.; Nowakiewicz, A.; Osińska, M.; Zięba, P. Diagnostic and Epidemiological Analysis of Trichophyton Benhamiae Infection on an Alpaca (Vicugna Pacos) Farm in Poland. *Vet. Ital.* **2021**, *57*, 319–327, doi:10.12834/VetIt.1967.10530.2.

122. Łagowski, D.; Gnat, S.; Nowakiewicz, A.; Trościańczyk, A. Real-time Pcr as an Alternative Technique for Detection of Dermatophytes in Cattle Herds. *Animals* **2021**, *11*, 7–9, doi:10.3390/ani11061662.

123. Larralde, M.; Gomar, B.; Boggio, P.; Abad, M.E.; Pagotto, B. Neonatal Kerion Celsi: Report of Three Cases. *Pediatr. Dermatol.* **2010**, *27*, 361–363, doi:10.1111/j.1525-1470.2010.01169.x.

124. Lavari, A.; Eidi, S.; Soltani, M. Molecular Diagnosis of Dermatophyte Isolates from Canine and Feline Dermatophytosis in Northeast Iran. *Vet. Med. Sci.* **2022**, *8*, 492–497, doi:10.1002/vms3.698.

125. Le Barzic, C.; Cmokova, A.; Denaes, C.; Arné, P.; Hubka, V.; Guillot, J.; Risco-Castillo, V. Detection and Control of Dermatophytosis in Wild European Hedgehogs (Erinaceus Europaeus) Admitted to a French Wildlife Rehabilitation Centre. *J. Fungi* **2021**, *7*, 1–13, doi:10.3390/jof7020074.

126. Lee, D.-W.; Yang, J.-H.; Choi, S.-J.; Won, C.-H.; Chang, S.-E.; Lee, M.-W.; Choi, J.-H.; Moon, K.-C.; Kim, M.-N. An Unusual Clinical Presentation of Tinea Faciei Caused by Trichophyton Mentagrophytes Var. Erinacei. *Pediatr. Dermatol.* **2011**, *28*, 210–212, doi:10.1111/j.1525-1470.2011.01391.x.

127. Li, D.-J.; Zhou, Y.-F.; Jing-Bo, L.; Mingliang, Z.; Yu-Mei, C.; Zeng-Min, M. Prevalence Investigation of Dermatophytes in Rabbits in Qingdao Region, China. *J. Anim. Vet. Adv.* **2012**, *11*, 883–885.

128. Limphoka, P.; Bunyaratavej, S.; Leeyaphan, C. Fingernail Onychomycosis Caused by Microsporum Canis in a Teenager. *Pediatr. Dermatol.* **2021**, *38*, 524–525, doi:10.1111/pde.14524.

129. Long, S.; Carveth, H.; Chang, Y.-M.; O'Neill, D.; Bond, R. Isolation of Dermatophytes from Dogs and Cats in the South of England between 1991 and 2017. *Vet. Rec.* **2020**, *187*, e87, doi:10.1136/vr.105957.

130. Longo, C.L.S.; Hercules, F.M.; Azevedo, F.S. de; Ferreira, A.L.P.; Orofino-Costa, R. Tinea Corporis Caused by Trichophyton Benhamiae: Report of the First Case Transmitted by Guinea Pig in Brazil. *An. Bras. Dermatol.* **2024**, *99*, 475–479, doi:10.1016/j.abd.2023.06.011.

131. Lopes, R.; Garcês, A.; Silva, A.; Brilhante-Simões, P.; Martins, Â.; Cardoso, L.; Duarte, E.L.; Coelho, A.C. Dermatophytosis in Companion Animals in Portugal: A Comprehensive Epidemiological Retrospective Study of 12 Years (2012–2023).

Microorganisms **2024**, *12*, 1–17, doi:10.3390/microorganisms12081727.

132. Lorch, J.M.; Minnis, A.M.; Meteyer, C.U.; Redell, J.A.; Paul White, J.; Kaarakka, H.M.; Muller, L.K.; Lindner, D.L.; Verant, M.L.; Shearn-Bochsler, V.; et al. The Fungus Trichophyton Redellii Sp. Nov. Causes Skin Infections That Resemble White-Nose Syndrome of Hibernating Bats. *J. Wildl. Dis.* **2015**, *51*, 36–47, doi:10.7589/2014-05-134.

133. Lysková, P.; Dobiáš, R.; Čmoková, A.; Kolařík, M.; Hamal, P.; Šmatláková, K.; Hušek, J.; Mencl, K.; Mallátová, N.; Poláčková, Z.; et al. An Outbreak of Trichophyton Quinckeanum Zoonotic Infections in the Czech Republic Transmitted from Cats and Dogs. *J. Fungi* **2021**, *7*, doi:10.3390/jof7090684.

134. Lyskova, P.; Hubka, V.; Petricakova, A.; Dobias, R.; Cmokova, A.; Kolarik, M. Equine Dermatophytosis Due to Trichophyton Bullosum, a Poorly Known Zoophilic Dermatophyte Masquerading as T. Verrucosum. *Mycopathologia* **2015**, *180*, 407–419, doi:10.1007/s11046-015-9931-0.

135. Marsicano, G.; de Araújo Roll, A.; Ferrelro, L.; Spanamberg, A.; Penter, C.D.; Cabral, J.N.H. Treatment of Dermatophytoses Caused by Microsporum Canis in Allouatta Guariba Primates. *Acta Sci. Vet.* **2010**, *38*, 449–452.

136. Martín-Penaranda, T.; Imbuluzqueta, J.M.L.; Gurrutxaga, M.A. Arthroderma Benhamiae in Patients with Guinea Pigs. *An Pediatr* **2019**, *90*, 51–52.

137. Maurice, M.N.; Kazeem, H.M.; Kwanashie, C.N.; Maurice, N.A.; Ngbede, E.O.; Adamu, H.N.; Mshelia, W.P.; Edeh, R.E. Equine Dermatophytosis: A Survey of Its Occurrence and Species Distribution among Horses in Kaduna State, Nigeria. *Scientifica (Cairo).* **2016**, *2016*, doi:10.1155/2016/6280646.

138. Mazur, M.; Lodyga, M.; Łańczak, A.; Adamski, Z. Majocchi's Granuloma (Granuloma Trichophyticum) in a Guinea Pig Owner: A Case Report and Literature Review. *J. Mycol. Med.* **2018**, *28*, 523–526, doi:10.1016/j.mycmed.2018.05.010.

139. Mesquita, J.R.; Vasconcelos-Nóbrega, C.; Oliveira, J.; Coelho, C.; Vala, H.; Fratti, M.; Arabatzis, M.; Velegraki, A.; Michel, M. Epizootic and Epidemic Dermatophytose Outbreaks Caused by Trichophyton Mentagrophytes from Rabbits in Portugal, 2015. *Mycoses* **2016**, *59*, 668–673, doi:10.1111/myc.12513.

140. Minnat, T.R. Epidemiological, Clinical and Laboratory Study of Canine Dermatophytosis in Baghdad Governorate, Iraq. *Iraqi J. Vet. Med.* **2019**, *43*, 183–196, doi:10.30539/iraqijvm.v43i1.489.

141. Minnat, T.R.; Khalf, J.M. Feline Dermatophytosis : Epidemiological, Clinical and Laboratory Features in Baghdad Governorate, Iraq. *Biochem. Cell. Arch.* **2019**, *19*, 4025–4033, doi:10.35124/bca.2019.19.2.4025.

142. Mochizuki, T.; Anzawa, K.; Bernales-Mendoza, A.M.; Shimizu, A. Case of Tinea Corporis Caused by a Terbinafine-Sensitive Trichophyton Indotineae Strain in a Vietnamese Worker in Japan. *J. Dermatol.* **2024**, 1–4, doi:10.1111/1346-8138.17463.

143. Mochizuki, T.; Kobayashi, H.; Takeda, K.; Anzawa, K.; Ishizaki, H. The First Human Cases of Americano-European Race of Arthroderma Benhamiae Infection in Japan. *Jpn. J.*

Infect. Dis. **2012**, *65*, 558–559, doi:10.7883/yoken.65.558.

144. Moreira, F.; Miranda, A.; Coelho, A.M.; Monteiro, J.; Coelho, A.C. Epidemiological Survey of Dermatophytosis in Meat Rabbits with Alopecia in Portugal. *World Rabbit Sci.* 2012, *20*, 43–48.

145. Moriello, K.A.; Leutenegger, C.M. Use of a Commercial QPCR Assay in 52 High Risk Shelter Cats for Disease Identification of Dermatophytosis and Mycological Cure. *Vet. Dermatol.* **2018**, *29*, 26–66, doi:10.1111/vde.12485.

146. Moriello, K.A.; Stuntebeck, R.; Mullen, L. Trichophyton Species and Microsporum Gypseum Infection and Fomite Carriage in Cats from Three Animal Shelters: A Retrospective Case Series. *J. Feline Med. Surg.* **2020**, *22*, 391–394, doi:10.1177/1098612X19846987.

147. Moriello, K.; Coyner, K.; Trimmer, A.; Newbury, S.; Kunder, D. Treatment of Shelter Cats with Oral Terbinafine and Concurrent Lime Sulphur Rinses. *Vet. Dermatol.* **2013**, *24*, doi:10.1111/vde.12069.

148. Morrell, J.; Stratman, E. Primary Care and Specialty Care Delays in Diagnosing Trichophyton Verrucosum Infection Related to Cattle Exposure. *J. Agromedicine* **2011**, *16*, 244–250, doi:10.1080/1059924X.2011.605715.

149. Moskaluk, A.; Vandewoude, S. Two Novel Species of Arthroderma Isolated from Domestic Cats with Dermatophytosis in the United States. *Med. Mycol.* **2022**, *60*, 1–10, doi:10.1093/mmy/myac001.

150. Murata, M.; Takahashi, H.; Takahashi, S.; Takahashi, Y.; Chibana, H.; Murata, Y.; Sugiyama, K.; Kaneshima, T.; Yamaguchi, S.; Miyasato, H.; et al. Isolation of Microsporum Gallinae from a Fighting Cock (Gallus Gallus Domesticus) in Japan. *Med. Mycol.* **2013**, *51*, 144–149, doi:10.3109/13693786.2012.701766.

151. Murmu, S.; Debnath, C.; Pramanik, A.K.; Mitra, T.; Jana, S.; Dey, S.; Banerjee, S.; Batabyal, K. Detection and Characterization of Zoonotic Dermatophytes from Dogs and Cats in and around Kolkata. *Vet. World* **2015**, *8*, 1078–1082, doi:10.14202/vetworld.2015.1078-1082.

152. Nardoni, S.; Rocchigiani, G.; Papini, R.A.; Veneziano, V.; Brajon, G.; Martini, M.; Salari, F.; Mancianti, F. Dermatophytosis in Donkeys (Equus Asinus) Due to Microsporum Racemosum, an Unusual Geophilic Agent. *Med. Mycol. Case Rep.* **2016**, *12*, 8–10, doi:10.1016/j.mmcr.2016.06.003.

153. Needle, D.B.; Gibson, R.; Hollingshead, N.A.; Sidor, I.F.; Marra, N.J.; Rothenheber, D.; Thachil, A.J.; Stanhope, B.J.; Stevens, B.A.; Ellis, J.C.; et al. Atypical Dermatophytosis in 12 North American Porcupines (Erethizon Dorsatum) from the Northeastern United States 2010–2017. *Pathogens* **2019**, *8*, doi:10.3390/pathogens8040171.

154. Neves, J.J.A.; Paulino, A.O.; Vieira, R.G.; Nishida, E.K.; Coutinho, S.D.A. The Presence of Dermatophytes in Infected Pets and Their Household Environment. *Arq. Bras. Med. Vet. e Zootec.* **2018**, *70*, 1747–1753, doi:10.1590/1678-4162-9660.

155. Newbury, S.; Moriello, K.A.; Kwochka, K.W.; Verbrugge, M.; Thomas, C. Use of

Itraconazole and Either Lime Sulphur or Malaseb Concentrate Rinse® to Treat Shelter Cats Naturally Infected with Microsporum Canis: An Open Field Trial. *Vet. Dermatol.* **2011**, *22*, 75–79, doi:10.1111/j.1365-3164.2010.00914.x.

156. Nikaein, D.; Yaghuti, P.; Sharifzadeh, A.; Khosravi, A.; Balal, A. Descriptive Epidemiology of Dermatophytosis in Rodents. *Vet. Med. Sci.* **2023**, *9*, 167–173, doi:10.1002/vms3.1044.

157. Nikkholgh, S.; Pchelin, I.M.; Zarei Mahmoudabadi, A.; Shabanzadeh-Bardar, M.; Gharaghani, M.; Sharifzadeh, A.; Mokhtari Hooyeh, M.; Mohammadi, R.; Nouripour-Sisakht, S.; Katiraee, F.; et al. Sheep Serve as a Reservoir of Trichophyton Mentagrophytes Genotype V Infection. *Med. Mycol.* **2023**, *61*, doi:10.1093/mmy/myad066.

158. Nobre, M. de O.; Negri Mueller, E.; Teixeira Tillmann, M.; da Silva Rosa, C.; Normanton Guim, T.; Vives, P.; Fernandes, M.; Martins Madrid, I.; Gevehr Fernandes, C.; Araújo Meireles, M.C. Disease Progression of Dermatophytic Pseudomycetoma in a Persian Cat. *Rev. Iberoam. Micol.* **2010**, *27*, 98–100, doi:10.1016/j.riam.2009.12.004.

159. Norrenberg, S.; Monod, M.; Christen-Zaech, S. Outbreak of Trichophyton Soudanense Causing Tinea Capitis in an Orphanage in Myanmar. *J. Med. Mycol.* **2020**, *30*, 101013, doi:10.1016/j.mycmed.2020.101013.

160. Oanţà, A.; Irimie, M. Tinea on a Tattoo. *Acta Dermatovenerologica Croat.* **2016**, *24*, 223–224.

161. Ogbonna, A.I.; Ogbonna, C.I.C.; Ogueri, S.C.; Nwadiaro, R.; Ishaku, H.L. Superficial Fungal Infections amongst Some Occupational Groups and HIV/AIDS Patients in Nigeria. *Asian J. Microbiol. Biotechnol. Environ. Sci.* **2014**, *16*, 7–10.

162. Oladzad, V.; Nasrollahi Omran, A.; Haghani, I.; Nabili, M.; Guillot, J.; Seyedmousavi, S.; Hedayati, M.T. Asymptomatic Colonization of Stray Dogs and Domestic Cats with Trichophyton Mentagrophytes II* in Northern Iran. *J. Med. Mycol.* **2024**, *34*, 101496, doi:10.1016/j.mycmed.2024.101496.

163. Oladzad, V.; Nasrollahi Omran, A.; Haghani, I.; Nabili, M.; Seyedmousavi, S.; Hedayati, M.T. Multi-Drug Resistance Trichophyton Indotineae in a Stray Dog. *Res. Vet. Sci.* **2024**, *166*, 105105, doi:10.1016/j.rvsc.2023.105105.

164. Orós, J.; Hernández, J.D.; Gallardo, J.; Lupiola, P.; Jensen, H.E. Dermatophytosis Caused by Trichophyton Spp. in a Tenerife Lizard (Gallotia Galloti): An Immunohistochemical Study. *J. Comp. Pathol.* **2013**, *149*, 372–375, doi:10.1016/j.jcpa.2012.11.245.

165. Özkanlar, Y.; Aktas, M.S.; Kirecci, E. Mycozoonosis Associated with Ringworm of Calves in Erzurum Province, Turkey. *Kafkas Univ. Vet. Fak. Derg.* **2009**, *15*, 141–144, doi:10.9775/kvfd.2008.85-G.

166. Pacifico Pereira, K.H.N.; Ritir Oliveira, E.L.; Baldissera Gonçalves, R.A.; Rolim, L.S.; das Neves Dias Neto, R.; Castilho, M.S.; Teixeira, C.R.; Rahal, S.C. Dermatophytosis Caused by Microsporum Canis in a Free-Living Maned Wolf (Chrysocyon Brachyurus). *Acta Sci. Vet.* **2018**, *46*, 1–4, doi:10.22456/1679-9216.86218.

167. Papini, R.; Nardoni, S.; Fanelli, A.; Mancianti, F. High Infection Rate of Trichophyton Verrucosum in Calves from Central Italy. *Zoonoses Public Health* **2009**, *56*, 59–64, doi:10.1111/j.1863-2378.2008.01157.x.

168. Paryuni, A.D.; Indarjulianto, S.; Untari, T.; Widyarini, S. Dermatophytosis in Cats: Clinical Signs and Identification of Etiological Agent. *Adv. Anim. Vet. Sci.* **2023**, *11*, 539–543, doi:10.17582/journal.aavs/2023/11.4.539.543.

169. Peano, A.; Arnoldi, S.; Čmoková, A.; Hubka, V. Re-Discovery of Trichophyton Bullosum in North Africa as a Cause of Severe Dermatophytosis in Donkeys. *Folia Microbiol. (Praha).* **2022**, *67*, 265–275, doi:10.1007/s12223-021-00930-9.

170. Peano, A.; Hubka, V.; Cavana, P.; Ottino, C.; Blandolino, M.; Molinar Min, A.R.; Pasquetti, M. Cases of Dermatophytosis Caused by Trichophyton Benhamiae Var. Luteum and T. Europaeum, Newly Described Dermatophytes within the T. Benhamiae Complex. *Vet. Dermatol.* **2022**, *33*, 440–445, doi:10.1111/vde.13082.

171. Phair, K.; Larsen, R.S.; Wack, R. Dermatophytosis (Trichophyton Mentagrophytes) in a Coquerel’s Sifaka (Propithecus Coquereli). *J. Zoo Wildl. Med.* **2011**, *42*, 759–762, doi:10.1638/2011-0033.1.

172. Phaitoonwattanakij, S.; Leeyaphan, C.; Bunyaratavej, S.; Chinhiran, K. Trichophyton Erinacei Onychomycosis: The First to Evidence a Proximal Subungual Onychomycosis Pattern. *Case Rep. Dermatol.* **2019**, *11*, 198–203, doi:10.1159/000501424.

173. Pieper, J.B.; Bowden, D.G.; Berger, D.J.; Noxon, J.O.; Grable, S.L.; Campbell, K.L. Trichophyton Mentagrophytes Complex: A Retrospective Study of 64 Dogs from the Central United States (1997–2020). *Vet. Dermatol.* **2023**, *34*, 310–317, doi:10.1111/vde.13160.

174. Piorunek, M.; Kubisiak-Rzepczyk, H.; Dańczak-Pazdrowska, A.; Trafas, T.; Walkowiak, J. Superficial Zoonotic Mycoses in Humans Associated with Cats. *J. Fungi* **2024**, *10*, doi:10.3390/jof10040244.

175. Polak, K.C.; Levy, J.K.; Crawford, P.C.; Leutenegger, C.M.; Moriello, K.A. Infectious Diseases in Large-Scale Cat Hoarding Investigations. *Vet. J.* **2014**, *201*, 189–195, doi:10.1016/j.tvjl.2014.05.020.

176. Prakash, V.A.; Rathish, R.L.; Deepa, P.M.; Bipin, K.C.; John, L. Comparative Efficacy of Three Stains for the Diagnosis of Canine Dermatophytosis. *Indian Vet. J.* **2023**, *100*, 27–31.

177. Pressanti, C.; Delverdier, M.; Iriart, X.; Morcel, F.; Cadiergues, M.C. A Case of Trichophyton Mentagrophytes Infection in a Fennec Fox (Vulpes Zerda). *Vet. Dermatol.* **2012**, *23*, 456–460, doi:10.1111/j.1365-3164.2012.01077.x.

178. Proverbio, D.; Perego, R.; Spada, E.; Bagnagatti De Giorgi, G.; Della Pepa, A.; Ferro, E. Survey of Dermatophytes in Stray Cats with and without Skin Lesions in Northern Italy. *Vet. Med. Int.* **2014**, *2014*, 2010–2013, doi:10.1155/2014/565470.

179. Qiao, X.; Hu, J.; Wu, D.; Wei, L.; Yang, Y.; Chen, J.; Mi, B.; Yang, S.Q. Isolation and Identification of Microsporum Canis from Asian Elephants (Elephas Maximus) in the

Chongqing Zoo, China. *J. Zoo Wildl. Med.* **2016**, *47*, 844–845, doi:10.1638/2014-0213.1.

180. Quintard, B.; Lohmann, C.; Lefaux, B. A CASE of TRYCHOPHYTON RUBRUM DERMATOPHYTOSIS in A PATAGONIAN SEA LION (OTARIA BYRONIA). *J. Zoo Wildl. Med.* **2015**, *46*, 621–623, doi:10.1638/2014-0214.1.

181. Rambozzi, L.; Meneguz, P.G.; Molinar Min, A.R.; Pasquetti, M.; Peano, A. Concurrent Chorioptic Mange and Dermatophytosis in Dairy Goats: A Case Report. *Vet. Sci.* **2022**, *9*, doi:10.3390/vetsci9120677.

182. Rhee, D.Y.; Kim, M.S.; Chang, S.E.; Lee, M.W.; Choi, J.H.; Moon, K.C.; Koh, J.K.; Choi, J.S. A Case of Tinea Manuum Caused by Trichophyton Mentagrophytes Var. Erinacei: The First Isolation in Korea. *Mycoses* **2009**, *52*, 287–290, doi:10.1111/j.1439-0507.2008.01556.x.

183. Rivaya, B.; Fernández-Rivas, G.; Cabañes, F.J.; Bielsa, I.; Castellá, G.; Wang, J.H.; Matas, L. Trichophyton Erinacei: An Emergent Pathogen of Pediatric Dermatophytosis. *Rev. Iberoam. Micol.* **2020**, *37*, 94–96, doi:10.1016/j.riam.2020.06.001.

184. Romano, C.; Massai, L.; Gallo, A.; Fimiani, M. Microsporum Gypseum Infection in the Siena Area in 2005-2006. *Mycoses* **2009**, *52*, 67–71, doi:10.1111/j.1439-0507.2008.01543.x.

185. Rostami, A.; Shirani, D.; Shokri, H.; Khosravi, A.R.; Daieghazvini, R.; Tootian, Z. Fungal Flora of the Hair Coat of Persian Squirrel (Sciurus Anomalus) with and without Skin Lesion in Tehran, Iran. *J. Mycol. Med.* **2010**, *20*, 21–25, doi:10.1016/j.mycmed.2009.11.004.

186. Salman, R.A.; Al-haddad, Z.A.A. Isolation and Identification of Aspergillus Fumigatus from Feline Respiratory Infection in Baghdad Province. *Syst. Rev. Pharm.* **2021**, *12*, 948–952.

187. Sanguansook, P.; Tuangpermsub, S.; Leelakarnsakul, B.; Phaisansomsuk, S.; Hunprasit, V.; Del Río, L.; Niyomtham, W.; Prapasarakul, N.; Sukhumavasi, W. Zoonotic Enteric Nematodes and Dermatophytes in Cat Cafés: An Investigation in the Bangkok Metropolitan Area, Thailand. *Vet. Sci.* **2024**, *11*, doi:10.3390/vetsci11080358.

188. Santana, A.E.; Taborda, C.P.; Filgueira, K.D.; Sellera, F.P.; Larsson, C.E.; Reche-Junior, A. Comparison of Carpet and Toothbrush Techniques for the Detection of Microsporum Canis in Cats. *J. Feline Med. Surg.* **2020**, *22*, 805–808, doi:10.1177/1098612X19880632.

189. Scarampella, F.; Zanna, G.; Peano, A.; Fabbri, E.; Tosti, A. Dermoscopic Features in 12 Cats with Dermatophytosis and in 12 Cats with Self-Induced Alopecia Due to Other Causes: An Observational Descriptive Study. *Vet. Dermatol.* **2015**, *26*, doi:10.1111/vde.12212.

190. Scarpa, M.A.; Etchecopaz, A.N.; Abrantes, R.A.; Mas, J.A.; Romero Núñez, C.; Miranda Contreras, L. Dermatophytosis Caused by Trichophyton Benhamiae in a Dog. *Vet. Dermatol.* **2021**, *32*, 297-e81, doi:10.1111/vde.12944.

191. Seker, E.; Dogan, N. Isolation of Dermatophytes from Dogs and Cats with Suspected Dermatophytosis in Western Turkey. *Prev. Vet. Med.* **2011**, *98*, 46–51,

doi:10.1016/j.prevetmed.2010.11.003.

192. Seyfarth, F.; Roediger, C.; Gräser, Y.; Erhard, M.; Burmester, A.; Elsner, P.; Hipler, U.-C. Case Report: Trichophyton Verrucosum Infection after Needlestick Injury with an Attenuated Live Vaccine against Cattle Ringworm. *Mycoses* **2011**, *54*, e870-6, doi:10.1111/j.1439-0507.2011.02015.x.

193. Sheetal, M.S.; Chandran, A.; Janus, A.; Sindhu, O.K.; Melepat, D.P.; Kaithathara, V.; Rathish, R.L. Dermatophytosis in Domestic Cats: Identification, and Treatment in an Indian Context. *Acta Trop.* **2024**, *255*, 107237, doi:10.1016/j.actatropica.2024.107237.

194. Shokri, H.; Khosravi, A.R. An Epidemiological Study of Animals Dermatomycoses in Iran. *J. Mycol. Med.* **2016**, *26*, 170–177, doi:10.1016/j.mycmed.2016.04.007.

195. Sidwell, R.U.; Chan, I.; Francis, N.; Bunker, C.B. Trichophyton Erinacei Kerion Barbae from a Hedgehog with Direct Osculatory Transfer to Another Person. *Clin. Exp. Dermatol.* **2014**, *39*, 38–40, doi:10.1111/ced.12197.

196. Sieklucki, U.; Oh, S.H.; Hoyer, L.L. Frequent Isolation of Arthroderma Benhamiae from Dogs with Dermatophytosis. *Vet. Dermatol.* **2014**, *25*, doi:10.1111/vde.12095.

197. Sierra-Maeda, K.Y.; Martínez-Hernández, F.; Arenas, R.; Boeta-Ángeles, L.; Martínez-Chavarría, L.C.; Rodríguez-Colín, S.F.; Xicohtencatl-Cortes, J.; Hernández-Castro, R. Tinea Corporis Intrafamilial Infection in Pets Due to Microsporum Canis. *Rev. Inst. Med. Trop. Sao Paulo* **2024**, *66*, 788–789, doi:10.1590/s1678-9946202466030.

198. Simsek, A. Investigation of Serum Beta-Defensin-1 Levels in Bovine Trichophytosis Cases. *Vet. World* **2021**, *14*, 2508–2511, doi:10.14202/vetworld.2021.2508-2511.

199. Sitterle, E.; Frealle, E.; Foulet, F.; Cabaret, O.; Cremer, G.; Guillot, J.; Delhaes, L.; Botterel, F. Trichophyton Bullosum: A New Zoonotic Dermatophyte Species. *Med. Mycol.* **2012**, *50*, 305–309, doi:10.3109/13693786.2011.605810.

200. Smagulova; Kukhar, Y. V.; Glotova, I.I.; Glotov, A.G.; Kim, A.S. First Record of Trichophyton Benhamiae Isolated from Domestic Cats in Russia. *Med. Mycol. Case Rep.* **2023**, *40*, 16–21, doi:10.1016/j.mmcr.2023.01.001.

201. Soares, F.O.; Rosado, I.R.; Sousa, M.V.C.; Dumont, C.F.; Bittar, J.F.F.; Martin, I.; Beletti, M.E.; Melo, R.T.; Alves, E.G.L. Osteomyelitis by Microsporum Canis and Staphylococcus Spp. in Cat (Felis Catus) – Case Report. *BMC Vet. Res.* **2024**, *20*, 1–8, doi:10.1186/s12917-024-03904-4.

202. Sos, E.; Molnar, V.; Lajos, Z.; Koroknai, V.; Gal, J. Successfully Treated Dermatomycosis in California Sea Lions (Zalophus Californianus). *J. Zoo Wildl. Med.* **2013**, *44*, 462–465.

203. Spanamberg, A.; Driemeier, D.; Sonne, L.; Ferreiro, L. Onychomycosis Caused by Malassezia Pachydermatis in a Dog. *Acta Sci. Vet.* **2019**, *47*, 47–50, doi:10.22456/1679-9216.94817.

204. Spanamberg, A.; Ravazzolo, A.P.; Araujo, R.; Tomazi, N.; Fuentes, B.; Ferreiro, L. Molecular Detection and Species Identification of Dermatophytes by SYBR-Green Real-

Time PCR in-House Methodology Using Hair Samples Obtained from Dogs and Cats. *Med. Mycol.* **2023**, *61*, doi:10.1093/mmy/myad047.

205. Spergser, J.; Neuhuber, T.; Haupt, H.; Kaltenegger, G.; Wittek, T. Agreement between Clinical Assessment and Laboratory Diagnosis of Ringworm in Calves at Auction Markets. *Animals* **2024**, *14*, 390, doi:10.3390/ani14030390.

206. St Clair, L.; Hopf, C.; Peters-Kennedy, J.; Mazulis, C.; Miller, J.; Scott, D.W.; Childs-Sanford, S. Regional Alopecia and Dermatitis Due to Lodderomyces Elongisporus in a North American Porcupine (Erethizon Dorsatum). *Vet. Dermatol.* **2021**, *32*, 188-e48, doi:10.1111/vde.12911.

207. Starace, M.; Carpanese, M.A.; Alessandrini, A.; Piraccini, B.M.; Patrizi, A.; Neri, I. Tinea Corporis Incognito Due to Microsporum Gypseum: Report of Eight Cases in Children. *Pediatr. Dermatol.* **2021**, *38*, 652–654, doi:10.1111/pde.14573.

208. Sun, P.L.; Mu, C.A.; Fan, C.C.; Fan, Y.C.; Hu, J.M.; Ju, Y.M. Cat Favus Caused by Microsporum Incurvatum Comb. Nov.: The Clinical and Histopathological Features and Molecular Phylogeny. *Med. Mycol.* **2014**, *52*, 276–284, doi:10.1093/mmy/myt023.

209. Sylvén, K.R.; Bergefur, A.L.; Jacobson, M.; Wallgren, P.; Selling, L.E. Dermatophytosis Caused by Trichophyton Mentagrophytes Complex in Organic Pigs. *Acta Vet. Scand.* **2023**, *65*, 1–8, doi:10.1186/s13028-023-00695-w.

210. Symoens, F.; Jousson, O.; Packeu, A.; Fratti, M.; Staib, P.; Mignon, B.; Monod, M. The Dermatophyte Species Arthroderma Benhamiae: Intraspecies Variability and Mating Behaviour. *J. Med. Microbiol.* **2013**, *62*, 377–385, doi:10.1099/jmm.0.053223-0.

211. Takahashi, C.; Asakura, R.; Chaya, A.; Ota, M.; Harada, K.; Inukai, T.; Nakamura, S.; Hata, Y.; Watanabe-Okada, E. Identification of Familial Infections Using Multilocus Microsatellite Typing in Tinea Corporis Due to Microsporum Canis: A Case Report. *Med. Mycol. J.* **2024**, *65*, 1–5, doi:10.3314/mmj.23-00013.

212. Tan, J.; Liu, X.; Gao, Z.; Yang, H.; Yang, L.; Wen, H. A Case of Tinea Faciei Caused by Trichophyton Benhamiae: First Report in China. *BMC Infect. Dis.* **2020**, *20*, 1–5, doi:10.1186/s12879-020-4897-z.

213. Tanabe, H.; Abe, N.; Anzawa, K. A Case of Tinea Corporis Caused by Trichophyton Benhamiae Var. Luteum from a Degu and Evolution of the Pathogen’s Taxonomy. *J. Fungi* **2023**, *9*, doi:10.3390/jof9111122.

214. Tartor, Y.H.; El-Neshwy, W.M.; Merwad, A.M.A.; Abo El-Maati, M.F.; Mohamed, R.E.; Dahshan, H.M.; Mahmoud, H.I. Ringworm in Calves: Risk Factors, Improved Molecular Diagnosis, and Therapeutic Efficacy of an Aloe Vera Gel Extract. *BMC Vet. Res.* **2020**, *16*, 1–15, doi:10.1186/s12917-020-02616-9.

215. Tel, O.Y.; Bozkaya, F.; Yigin, A.; Gürbilek, S.E.; Keskin, O. Isolation and Molecular Characterization of Bovine Trichophyton Verrucosum Strains Based on Sequence Analysis of Internal Transcribed Spacer Region (ITS1) and Microsatellite Loci. *Pak. Vet. J.* **2018**, *38*, 189–193, doi:10.29261/pakvetj/2018.040.

216. Tobeigei, F.H.; Joseph, M.R.; Al-Hakami, A.; Hamid, M.E. Microsporum Gypseum

Infection Among Two Related Families With a Zoonotic Aspect: A Prospective Case Series. *Cureus* **2023**, *15*, 1–11, doi:10.7759/cureus.51402.

217. Tresamol, P. V.; Saseendranath, M.R.; Subramanian, H.; Pillai, U.N.; Mini, M.; Ajithkumar, S. Identification of Dermatophilus Congolensis from Lower Leg Dermatitis of Cattle in Kerala, India. *OIE Rev. Sci. Tech.* **2015**, *34*, 849–854, doi:10.20506/rst.34.3.2400.

218. Tsai, Y.J.; Chung, W.C.; Wang, L.C.; Ju, Y. Ten; Hong, C.L.; Tsai, Y.Y.; Li, Y.H.; Wu, Y.L. The Dog Mite, Demodex Canis: Prevalence, Fungal Co-Infection, Reactions to Light, and Hair Follicle Apoptosis. *J. Insect Sci.* **2011**, *11*, 1–13, doi:10.1673/031.011.7601.

219. Tuteja, F.C.; Patil, N. V; Narnaware, S.D.; Nagarajan, G.; Dahiya, S.S. Camel Dermal Mycoses Caused by Dermatophytes. *J. Camel Pract. Res.* **2013**, *20*, 157–165.

220. Uhrlaß, S.; Mey, S.; Storch, S.; Wittig, F.; Koch, D.; Krüger, C.; Nenoff, P. Nannizzia Incurvata as a Rare Cause of Favus and Tinea Corporis in Cambodia and Vietnam. *Indian J. Dermatol. Venereol. Leprol.* **2018**, *87*, 515–521, doi:10.4103/ijdvl.IJDVL_954_18.

221. Umitzhanov, M.; Abdiramanova, B.; Abutalip, A.; Bakirov, N.; Sarimbekova, S. Comparative Assessment of Regulated Methods and PCR in the Diagnosis of Trichophytosis in Veterinary Mycology. *Open Vet. J.* **2023**, *13*, 1614–1622, doi:10.5455/OVJ.2023.v13.i12.11.

222. Vanam, H.P.; Mohanram, K.; K, S.R.R.; Poojari, S.S.; P.R, A.; Kandi, V. First Report of Concomitant Tinea Faciei and Pityriasis Folliculorum: A Dermatomicrobiological Rarity. *Cureus* **2018**, *10*, doi:10.7759/cureus.3017.

223. Vanam, H.P.; Mohanram, K.; Reddy, K.S.R.; Rengasamy, M.; Rudramurthy, S.M. Naive Tinea Corporis et Cruris in an Immunocompetent Adult Caused by a Geophile Nannizzia Gypsea Susceptible to Terbinafine–Rarity in the Current Scenario of Dermatophytosis in India. *Access Microbiol.* **2019**, *1*, doi:10.1099/acmi.0.000022.

224. Vanderwolf, K.J.; Campbell, L.J.; Goldberg, T.L.; Blehert, D.S.; Lorch, J.M. Skin Fungal Assemblages of Bats Vary Based on Susceptibility to White-Nose Syndrome. *ISME J.* **2021**, *15*, 909–920, doi:10.1038/s41396-020-00821-w.

225. Veeraselvam, M.; Selvaraj, P.; Jayalakshmi, K.; Yogeshpriya, S.; Venkatesan, M.; Premalatha, N.; Saravanan, M.; Ramkumar, P.K. Dermatophytosis in a Nomadic Circus Camel and Its Management with Miconazole Therapy. *J. Camel Pract. Res.* **2020**, *27*, 367–370, doi:10.5958/2277-8934.2020.00053.3.

226. Veraldi, S.; Genovese, G.; Peano, A. Tinea Corporis Caused by Trichophyton Equinum in a Rider and Review of the Literature. *Infection* **2018**, *46*, 135–137, doi:10.1007/s15010-017-1067-3.

227. Veraldi, S.; Guanziroli, E.; Schianchi, R. Epidemic of Tinea Corporis Due to Trichophyton Mentagrophytes of Rabbit Origin. *Pediatr. Dermatol.* **2012**, *29*, 392–393, doi:10.1111/j.1525-1470.2012.01782.x.

228. Walsh, A.L.; Merchan, N.; Harper, C.M. Hedgehog-Transmitted Trichophyton Erinaceid

Causing Painful Bullous Tinea Manuum. *J. Hand Surg. Am.* **2021**, *46*, 430.e1-430.e3, doi:10.1016/j.jhsa.2020.06.015.

229. Wang, F.Y.; Sun, P.L. Tinea Blepharo-Ciliaris in a 13-Year-Old Girl Caused by Trichophyton Benhamiae. *J. Mycol. Med.* **2018**, *28*, 542–546, doi:10.1016/j.mycmed.2018.05.002.

230. Wang, L.; Fu, J.; Cai, G.; Cheng, X.; Zhang, D.; Shi, S.; Zhang, Y. Rapid and Visual RPA-Cas12a Fluorescence Assay for Accurate Detection of Dermatophytes in Cats and Dogs. *Biosensors* **2022**, *12*, doi:10.3390/bios12080636.

231. Watanabe, M.; Tsuchihashi, H.; Ogawa, T.; Ogawa, Y.; Komiyama, E.; Hirasawa, Y.; Hiruma, M.; Kano, R.; Ikeda, S. Microsporum Canis Infection in a Cat Breeder Family and an Investigation of Their Breeding Cats. *Med. Mycol. J.* **2022**, *63*, 139–142, doi:10.3314/mmj.22-00015.

232. Watanabe, R.; Furuta, H.; Ueno, Y.; Nukada, T.; Niwa, H.; Shinyashiki, N.; Kano, R. First Isolation of Trichophyton Bullosum from a Horse with Dermatophytosis in Japan. *Med. Mycol. Case Rep.* **2021**, *32*, 81–83, doi:10.1016/j.mmcr.2021.04.004.

233. Wei, S.; Wang, H.; Li, A.; Yuan, C. Kerion Celsi Caused by Microsporum Gypseum in a Chinese Child, a Case Report. *Med. (United States)* **2022**, *101*, E28936, doi:10.1097/MD.0000000000028936.

234. Weishaupt, J.; Kolb-Mäurer, A.; Lempert, S.; Nenoff, P.; Uhrlaß, S.; Hamm, H.; Goebeler, M. A Different Kind of Hedgehog Pathway: Tinea Manus Due to Trichophyton Erinacei Transmitted by an African Pygmy Hedgehog (Atelerix Albiventris). *Mycoses* **2014**, *57*, 125–127, doi:10.1111/myc.12113.

235. Westhoff, D.K.; Kloes, M.-C.; Orveillon, F.X.; Farnow, D.; Elbers, K.; Mueller, R.S. Treatment of Feline Dermatophytosis with an Inactivated Fungal Vaccine. *Open Mycol. J.* **2010**, *4*, 10–17, doi:10.2174/1874437001004010010.

236. White, S.D.; Affolter, V.K.; Molinaro, A.M.; Depenbrock, S.M.; Chigerwe, M.; Heller, M.C.; Rowe, J.D. Skin Disease in Goats (Capra Aegagrus Hircus): A Retrospective Study of 358 Cases at a University Veterinary Teaching Hospital (1988–2020). *Vet. Dermatol.* **2022**, *33*, 227-e64, doi:10.1111/vde.13052.

237. White, S.D.; Sanchez-Migallon Guzman, D.; Paul-Murphy, J.; Hawkins, M.G. Skin Diseases in Companion Guinea Pigs (Cavia Porcellus): A Retrospective Study of 293 Cases Seen at the Veterinary Medical Teaching Hospital, University of California at Davis (1990-2015). *Vet. Dermatol.* **2016**, *27*, doi:10.1111/vde.12348.

238. Yamada, S.; Anzawa, K.; Mochizuki, T. Molecular Epidemiology of Microsporum Canis Isolated from Japanese Cats and Dogs, and from Pet Owners by Multilocus Microsatellite Typing Fragment Analysis. *Jpn. J. Infect. Dis.* **2022**, *75*, 105–113, doi:10.7883/yoken.JJID.2020.809.

239. Yang, Y.P.; Sheng, P.; Liu, Z.; Li, W.; Wang, J. Di; Huang, W.M.; Fan, Y.M. Kerion and Tinea Corporis Caused by Rabbit-Derived Trichophyton Interdigitale in Three Siblings and One Consulting Doctor Using β-Tubulin Gene to Identify the Pathogen.

Mycopathologia **2016**, *181*, 539–546, doi:10.1007/s11046-016-9998-2.

240. Yildirim, M.; Cinar, M.; Ocal, N.; Yagci, B.B.; Askar, S. Prevalence of Clinical Dermatophytosis and Oxidative Stress in Cattle. *J. Anim. Vet. Adv.* **2010**, *9*, 1978–1982, doi:10.3923/javaa.2010.1978.1982.

241. Yin, B.; Ran, X.; Zhang, C.; Xie, Z.; Ran, Y.; Fu, L.; Pradhan, S. Tinea Incognito Infection with Trichophyton Erinacei from a Pet Hedgehog. *Br. J. Dermatol.* **2020**, *183*, e92, doi:10.1111/bjd.19206.

242. Yin, B.; Xiao, Y.; Ran, Y.; Kang, D.; Dai, Y.; Lama, J. Microsporum Canis Infection in Three Familial Cases with Tinea Capitis and Tinea Corporis. *Mycopathologia* **2013**, *176*, 259–265, doi:10.1007/s11046-013-9685-5.

243. Zeng, J.; Wang, S.; Guo, L.; Lv, S.; Shan, B.; Liu, Z.; Li, F. Pediatric Tinea Capitis in Jilin Province: Analyzing Previous Results from a New Perspective. *Mycopathologia* **2023**, *188*, 515–522, doi:10.1007/s11046-023-00718-0.

244. Zeng, X.; Zheng, Q.; Chi, X. A Case of Trichophyton Mentagrophytes Infection in Rabbits Accompanied by Farm Staff Infection in China. *Kafkas Univ. Vet. Fak. Derg.* **2017**, *23*, 497–501, doi:10.9775/kvfd.2016.16685.

245. Zhang, H.; Ran, Y.; Liu, Y.; Zhang, R.; Lin, X.; Yan, W.; Dai, Y. Arthroderma Vanbreuseghemii Infection in Three Family Members with Kerion and Tinea Corporis. *Med. Mycol.* **2009**, *47*, 539–544, doi:10.1080/13693780802644627.

246. Zhang, Y.; Luo, W.; Tang, C.; Sybren de Hoog, G.; Lu, H.; Jiang, Y. Possible Rabbit Breeders' Trichophyton Mentagrophytes Infection Characterized by Majocchi's Granuloma in Immunocompetent Host: Case Report. *Med. Mycol. Case Rep.* **2019**, *26*, 19–22, doi:10.1016/j.mmcr.2019.09.007.

247. Zheng, D.; Liang, T.; Wu, W.; Al-Odaini, N.; Pan, K.; Huang, L.; Huang, G.; Tang, L.; Li, X.; He, S.; et al. The Epidemiology of Tinea Capitis in Guangxi Province, China. *Mycopathologia* **2023**, *188*, 489–496, doi:10.1007/s11046-023-00762-w.

248. Zheng, Y.Y.; Li, Y.; Chen, M.Y.; Mei, Q.Y.; Zhang, R.Z. Majocchi's Granuloma on the Forearm Caused by Trichophyton Tonsurans in an Immunocompetent Patient. *Ann. Clin. Microbiol. Antimicrob.* **2020**, *19*, 1–4, doi:10.1186/s12941-020-00382-y.

249. Zhi, H.L.; Xia, X.J.; Shen, H.; Lv, W.W.; Zhong, Y.; Sang, B.; Li, Q.P.; Liu, Z.H. Trichoscopy for Early Diagnosis and Follow-up of Pet-Related Neonatal Tinea Capitis. *Mycopathologia* **2023**, *188*, 571–575, doi:10.1007/s11046-023-00709-1.

250. Ziglioli, V.; Panciera, D.L.; Leroith, T.; Wiederhold, N.; Sutton, D. Invasive Microsporum Canis Causing Rhinitis and Stomatitis in a Cat. *J. Small Anim. Pract.* **2016**, *57*, 327–331, doi:10.1111/jsap.12471.
